# P05.45. A whole systems approach to the study of Ayurveda for cancer survivorship: results from a qualitative investigation

**DOI:** 10.1186/1472-6882-12-S1-P405

**Published:** 2012-06-12

**Authors:** A Dhruva, F Hecht, J Weaver, T Kaptchuk, V Lad, S Adler

**Affiliations:** 1 University of California, San Francisco, San Francisco, USA; 2 Harvard Medical School, Boston, USA; 3 Ayurvedic Institute, Albuquerque, USA

## Purpose

To describe an approach to the development of manualized protocols for Ayurveda as a whole system of medicine; to characterize an Ayurvedic perspective on the pathophysiology, etiologies, and supportive treatments for an allopathic diagnosis of cancer.

## Methods

Hour-long, digitally recorded interviews were conducted with experienced Ayurvedic clinicians from both the USA and India. Eligible clinicians had an advanced degree in Ayurveda and clinical experience with a minimum of 50 patients with a diagnosis of cancer. All interviews were transcribed verbatim and coded for major themes using principles of qualitative content analysis. At least two investigators coded each interview. Variations in interpretation were reconciled through refined definitions and recoding.

## Results

Ten participants, with an average of 23 years of clinical experience, were interviewed. Several themes emerged from the data: (1) The Ayurvedic description of the pathophysiology of cancer uses traditional concepts translated into the modern context. ( 2) Although the allopathic treatment of cancer is considered necessary, from an Ayurvedic perspective, it results in degeneration and depletion. (3) In cases where Allopathic treatment has stopped working or is not feasible, an Ayurvedic approach focusing on strengthening digestion, burning toxins, reducing growth, and improving tissue metabolism is viewed as useful. (4) An Ayurvedic approach to cancer supportive care focuses on restoring equilibrium, building mental and physical strength, and rejuvenation. Qualitative data were used to develop a study manual focusing on an individualized, multi-modality Ayurvedic intervention for breast cancer survivorship.

## Conclusion

A methodology for whole systems research that starts with a qualitative approach can be used to develop a manualized Ayurvedic intervention. Although derived from an ancient, but living tradition, Ayurvedic medicine offers a unique perspective on the modern allopathic diagnosis of cancer that emphasizes restoring wholeness and integrity to the mind and body; using natural remedies; including a focus on emotional health; and instituting prevention strategies.

